# Attitudes, Motivators, and Barriers to Emergency Preparedness Using the 2016 Styles Survey

**DOI:** 10.1177/1524839918794940

**Published:** 2018-08-20

**Authors:** Judy Kruger, Brenda Chen, Suzanne Heitfeld, Lauren Witbart, Crystal Bruce, Dana L. Pitts

**Affiliations:** 1 Centers for Disease Control and Prevention, Atlanta, GA, USA

**Keywords:** disaster and emergency response, health research, resource development

## Abstract

This study assessed adults’ perceptions toward preparedness to better inform emergency planning efforts for households and communities. The 2016 *Styles*, an Internet panel survey, was used to assess emergency preparedness competencies. Descriptive analyses were performed to describe the sociodemographic factors by preparedness status. Multivariable logistic regressions were used to examine the association between perceived preparedness and characteristics associated with preparedness attitudes, motivators, and barriers. Approximately 40% of adults surveyed reported that they were prepared for emergencies. The main motivator for those prepared was awareness of local disasters (38.9%), and a leading barrier was confusion about how to plan for the unknown (23.7%). Those prepared were more likely to have the right supplies (adjusted odds ratio [AOR] = 1.25, 95% confidence interval [CI] = [1.05, 1.50]), discuss emergency plans (AOR = 1.21, 95% CI = [1.02-1.42]), and act before an emergency occurred (AOR = 1.35, 95% CI = [1.15, 1.59]), compared with adults who did not report being prepared. Results from this research indicate that identifying motivation to prepare for emergencies can contribute to public health disaster planning. Preparation is a critical step that allows the community and its citizens to be more equipped to function during and after a disaster.

## Introduction

Experience with public health emergencies confirms that every community in the United States must be ready for unpredictable events, such as pandemics, national disasters, acts of terrorism, or chemical or radiological releases. Most important, individuals and households that comprise communities must have knowledge, tools, and confidence to take appropriate action when needed. Despite our national investment and experience with disasters, American household preparedness has improved only modestly since 2003 (Petkiva et al., 2016; U.S. Department of Homeland Security, 2007). To build community disaster resilience, government and emergency management agencies are encouraged to collaborate to develop resilience-building activities and programs (e.g., where, when, and what to do before a local disaster occurs) based on jurisdictions preparedness trends and patterns (McCormick, Fifolt, Mercer, Pevear, & Wilson, 2017). Tracking and assessment of household preparedness and individual perceptions of preparedness using timely data collected through the Internet can be useful to direct investments (Petkiva et al., 2016). Characterizing individual and household preparedness may help researchers understand how the public’s attitudes correspond with their own knowledge, motivation, and ability to prepare. Moreover, these insights may help communities better target investments to improve readiness and resilience.

### Connecting Individual and Community Resilience

The term *community resilience* encompasses the association between individuals taking action to reduce their risk during a disaster and the resiliency of a community (Community and Regional Resilience Institute, 2013; Levac, Toal-Sulivan, & O’Sullivan, 2011; Wolf-Fordham, Curtin, Maslin, Bandini, & Hamad, 2015). People who are resilient and/or prepared may be less likely to experience health hazards during emergencies and more likely to reestablish daily activities after a disaster (Petkiva et al., 2016). National data suggest that people trained in, and experienced with disasters, are more resilient and that community resilience (including actions that promote national preparedness) affects community-level preparedness (Butts, Beaujean, Richardus, & Voeten, 2014; Gowan, Kirk, & Sloan, 2014; Labaka, Hernantes, Rich, & Sarriegi, 2013). Examples of individual actions that promote national preparedness include identifying communication channels as information resources, attending tabletop exercises trainings related to enhancing preparedness capabilities, and participating in stakeholder meetings with organizations and sectors working to improve community preparedness (Matthews et al., 2005).

### Importance of Understanding Public Perception and Disaster Preparedness

Household preparedness efforts have shifted over time from a focus on adequate supply of water, food, and medicine to a community resilience approach that emphasizes a person’s risk perception as a means of better coping with the consequences of a hazard (Federal Emergency Management Agency [FEMA], 2013). Research suggests that people’s perceptions about preparedness and their *self-efficacy* (i.e., an individual’s confidence in their ability to perform a behavior) affects their likelihood to take action to prepare for an emergency (Adams, Rivard, & Eisenman, 2017; Thomas, Leander-Griffith, Harp, & Cioffi, 2015).

Behavioral theories and models have been applied to disaster and emergency preparedness to identify constructs that may be useful to guide public health professionals and emergency managers in developing and implementing interventions before a disaster happens (Ejeta, Ardalan, & Paton, 2015). Despite the advancement in theoretical models, many theories are used to tailor interventions and target individuals based on their readiness for change, and recognize barriers to action, such as socioeconomic status, physical security, or lack of knowledge (Community and Regional Resilience Institute, 2013). One theoretical model, called disaster preparedness behavior (DPB), which is based on the theory of planned behavior, suggests that prepared adults are influenced by their perception of control over the DPB (Najafi, Ardalan, Akbarisari, Noorbala, & Elmi, 2017). Although the demographic determinants of DPB do not provide a complete description of the process that underlie the formation of intentions related to disaster preparedness, general education and knowledge about disasters can influence preparedness behavior (Najafi, Ardalan, Akbarisari, Noorbala, & Jabbari, 2015). This article builds on prior research and examines the association between perceived preparedness and characteristics associated with preparedness attitudes, motivators, and barriers among adults participating in the 2016 *Styles* survey.

## Methods

The data used in this study were obtained from the fall wave of the 2016 *Styles* survey conducted by Porter Novelli (Washington, D.C.), and assessed attitudes, motivators, and barriers toward emergency preparedness. *Styles* was administered annually to a nationwide sample of noninstitutionalized U.S. adults aged ⩾18 years. Porter Novelli draws from GfK’s KnowledgePanel® (an online panel), using probability-based sampling (GfK, 2014). The panel has been continuously replenished to maintain approximately 55,000 panelists.

Volunteer participants were recruited through quota sampling of households using a consumer mail panel that represents a range of sociodemographic characteristics and whether or not a respondent had Internet access prior to joining the panel. The survey was administered seasonally, and respondents were given a small gift (e.g., received reward points worth approximately $5) and an opportunity to enter a sweepstake in return for their participation. In 2016, 4,495 adults were sampled for the *Styles* survey (September 19 to October 3, 2016), and 3,544 adults completed the survey. The overall response rate was 78.8%. Centers for Disease Control and Prevention licensed the results of the 2016 *Styles* survey postcollection from Porter Novelli. The deidentified data used for this study were exempt from institutional review board review because it was deemed a secondary analysis and not involving human subjects. The Paper Work Reduction Act did not apply.

### Measures

#### Sociodemographic Variables

The *Styles* survey asked respondents to provide information on their sex, age (coded into categories: 18-24, 25-44, 45-64, and ⩾65 years), race/ethnicity (e.g., non-Hispanic White, non-Hispanic Black, Hispanic, non-Hispanic other), educational attainment (less than high school, high school graduate, some college, college graduate), marital status (currently married, previously married, never married), children in the household (yes, no), annual household income (coded into categories: <$25,000, $25,000-$39,999, $40,000-$59,999, ⩾$60,000), census region (Northeast, Midwest, South, West), and metropolitan statistical area status (nonmetro, metro).

#### Perceived Preparedness

Perceived preparedness was assessed by the question “How prepared are you for emergencies (i.e., *you have plans in place, you have talked about your plans with your family*, and/or *you have the supplies necessary to carry out the plans*)?” Response options were *not at all, a little likely, somewhat likely*, and *very likely*. Respondents who selected *somewhat* or *very prepared* were classified as being prepared.

#### Preparedness Attitudes

Respondents’ preparedness attitudes were assessed by the question “What does emergency preparedness for disasters and disease outbreaks mean to you?” Response options included *having the right emergency supplies, knowing what to do during an emergency, discussing plans in case of emergencies, being able to react to an unplanned event, acting before an emergency occurs*, and *preventing harm to self and family*. Respondents were asked to select as many strategies as appropriate. The response items were not mutually exclusive, and any possible combinations of these factors could be selected.

### Motivators

Motivating factors for being prepared were assessed by the question “What would make you more likely to prepare for emergencies?” Response options included *nothing, I am already prepared, personal experience with a disaster, information about possible local disasters, increased likelihood of a local disaster, information about consequences (losses)*, and *if I receive a discount to buy basic supplies*. Respondents were asked to select as many items as applied to them. The response items were not mutually exclusive, and any possible combinations of these factors could be selected.

### Barriers

Barriers to being prepared were assessed by an open-field question “What makes it difficult for you to prepare for emergencies?” Response options included *talking to my family about it is hard, planning for the unknown is confusing, I don’t know where to begin or what to do, I can’t afford to buy supplies*, and *I don’t think it is important where I live*. Respondents were asked to select as many strategies as appropriate. The response items were not mutually exclusive, and any possible combinations of these factors could be selected.

### Statistical Analysis

*Styles* data were weighted to match the U.S. Current Population Survey proportions for sex, age, household income, race/ethnicity, household size, education level, census region, metro status, and whether or not a respondent had Internet access prior to joining the panel. Weighted frequency analyses were run to characterize the sociodemographic adult sample by respondents’ self-reported status (e.g., prepared and not prepared). Sociodemographic differences by perceived preparedness status were assessed by chi-square tests. Point estimates were calculated for adults who reported that they were prepared by attitudes, motivators, and barriers to preparing for an emergency. Multivariate logistic regressions were used to assess preparedness correlates among adults who reported being prepared, adjusted for sex, age, and race/ethnicity. All analyses were conducted using SAS version 9.3 Proc Surveyfreq and Proc Surveylogistic (SAS Institute, Inc., Cary, NC).

## Results

In terms of perceived preparedness, approximately 40.0% of adults (numerator: 1,385; denominator: 3,463) responding to the study survey reported being prepared (Table 1). Among those who reported being prepared, statistically significant differences were found by age, education, marital status, and census region (*p* < .05). Within-group comparisons found that a higher proportion of respondents ⩾65 years (49.0%) perceived that they were prepared, compared with those aged 18 to 24 years (33.7%). By education, a higher proportion of adults with a high school education (42.8%) perceived that they were prepared, compared with those with less than a high school education (33.1%). By marital status, a higher proportion of adults who were previously married (43.5%) perceived that they were prepared, compared with those never married (33.4%). By census region, a higher proportion of adults who resided in the South (44.9%) perceived that they were prepared, compared with those living in the Midwest (35.6%).

**Table 1:** Prevalence of Reporting Household Emergency Preparedness Among Adults (⩾18 years), by Perceived Preparedness Status—*Styles* Survey, 2016

Characteristic	Perception of Household Emergency Preparedness (*n* = 3,463)						
	Not Prepared^a^			Prepared^b^			
	*n* (Unweighted)^c^	*n* (Weighted)^d^	%^e^ [95% CI]	*n* (Unweighted)^c^	*n* (Weighted)^d^	%^e^ [95% CI]	Chi-Square
Sex							
Men	985	973	58.1 [55.4, 60.9]	764	700	41.9 [39.1, 44.6]	3.27
Women	1,032	1,103	61.7 [59.0, 64.4]	682	684	38.3 [35.6, 41.0]	
Age (years)							
18-24	316	489	66.3 [61.5, 71.1]	155	249	33.7 [28.9, 38.5]	34.08*
25-44	434	549	63.9 [59.8, 68.1]	235	310	36.1 [31.9, 40.2]	
45-64	594	547	60.6 [57.2, 64.0]	398	355	39.4 [36.0, 42.8]	
⩾65	673	491	51.0 [47.8, 54.2]	658	471	49.0 [45.8, 52.2]	
Race/ethnicity							
Non-Hispanic White	1,482	1,340	59.1 [56.9, 61.3]	1,087	928	40.9 [38.7, 43.1]	5.69
Non-Hispanic Black	182	223	55.9 [49.8, 62.1]	152	176	44.1 [37.9, 50.2]	
Hispanic	257	339	63.7 [58.2, 69.1]	147	193	36.3 [30.9, 41.8]	
Non-Hispanic other	96	172	66.2 [57.1, 75.4]	60	88	33.8 [24.6, 42.9]	
Education							
<High school	143	274	66.9 [60.1, 73.6]	81	136	33.1 [26.4, 39.9]	10.69*
High school	638	592	57.2 [53.8, 60.6]	472	443	42.8 [39.4, 46.2]	
Some college	578	559	57.5 [53.9, 61.0]	461	414	42.5 [39.0, 46.1]	
College graduate	658	650	62.4 [59.0, 65.7]	432	392	37.6 [34.3, 41.0]	
Marital status							
Currently married	1,059	1,077	57.3 [54.7, 59.8]	850	804	42.7 [40.2, 45.3]	19.40*
Previously married^f^	369	296	56.5 [51.8, 61.2]	301	228	43.5 [38.8, 48.2]	
Never married^g^	589	702	66.6 [62.8, 70.3]	295	352	33.4 [29.7, 37.2]	
Children in household							
Yes	1,562	1,473	59.8 [57.5, 62.0]	1,143	992	40.2 [38.0, 42.5]	0.12
No	455	602	60.5 [56.6, 64.4]	303	392	39.5 [35.6, 43.4]	
Annual household income							
<$25,000	367	361	61.3 [56.5, 66.1]	246	228	38.7 [33.9, 43.5]	1.64
$25,000-$39,999	341	286	62.4 [57.6, 67.2]	239	172	37.6 [32.8, 42.4]	
$40,000-$59,999	347	324	58.9 [54.3, 63.4]	253	226	41.1 [36.6, 45.7]	
⩾$60,000	962	1,104	59.3 [56.6, 62.0]	708	758	40.7 [38.0, 43.4]	
U.S. census region							
Northeast	383	376	60.1 [55.6, 64.6]	255	250	39.9 [35.4, 44.4]	16.78*
Midwest	512	477	64.4 [60.5, 68.2]	305	264	35.6 [31.8, 39.5]	
South	659	705	55.1 [51.8, 58.4]	584	575	44.9 [41.6, 48.2]	
West	463	517	63.6 [59.5, 67.7]	302	296	36.4 [32.3, 40.5]	
Metropolitan statistical area							
Nonmetro	291	290	55.8 [50.7, 60.9]	234	230	44.2 [39.1, 49.3]	3.14
Metro	1,726	1,785	60.7 [58.6, 62.8]	1,212	1,155	39.3 [37.2, 41.4]	
Overall	2,017	2,075	60.0 [58.0, 61.9)	1,446	1,385	40.0 [38.1, 42.0]	

NOTE: Data were only analyses for each major sociodemographic characteristic. Chi-square tests were used to calculate *p* values for unadjusted difference in the percent of those who reported being prepared and not prepared separately for each individual sociodemographic characteristic. CI = confidence interval.

a Among those who self-reported that they were not at all or a little prepared for the question “How prepared are you for emergencies (e.g., you have plans in place, you have talked about your plans with your family, and/or you have the supplies necessary to carry out the plans)?” ^b^Among those who self-reported that they were somewhat or very prepared for the question “How prepared are you for emergencies (e.g., you have plans in place, you have talked about your plans with your family, and/or you have the supplies necessary to carry out the plans)?” ^c^Unweighted sample size of those who self-reported their preparedness status. ^d^Weighted sample size of those who self-reported their preparedness status. ^e^Percentage was weighted to match the U.S. Current Population Survey proportions for sex, age, household income, race/ethnicity, household size, education level, census region, metro status, and whether or not a respondent had Internet access. ^f^Includes divorced, widowed, and separated persons. ^g^Includes persons who were never married and members of unmarried couples.

* Significant at *p* < .05.

Seventy-six percent of adults who were prepared reported that they know what to do during an emergency (Table 2). The top three motivating factors reported to bolster preparedness include an increased likelihood of a local disaster (38.9%; 95% CI = [36.0, 41.9]), receiving information about a possible local disaster (36.0%; 95% CI = [33.1, 38.9]), and a personal experience with a disaster (23.5%; 95% CI = [20.9-26.0]). The top three barriers to preparing for an emergency were confusion on how to plan for the unknown (23.7%; 95% CI = [21.2, 26.3]), inability to buy supplies (16.7%; 95% CI = [14.4, 19.1]), and not thinking it is important where they live (10.9%; 95% CI = [8.9, 12.8]).

**Table 2:** Perceived Preparedness Attitudes, Motivating Factors, and Barriers Among Adults (⩾18 years) Who Reported Being Prepared (*N* = 1,446)—*Style*s Survey, 2016

Characteristics	*n* (Weighted)^a^	%^b^ [95% CI]
Attitudes^c^		
Knowing what to do during an emergency	1,057	76.3 [73.5, 79.1]
Having the right emergency supplies	946	68.3 [65.3, 71.3]
Being able to react to an unplanned event	903	65.2 [62.2, 68.3]
Discussing plans in case of emergencies	802	57.9 [54.9, 61.0]
Preventing harm to myself and family	784	56.6 [53.6, 59.7]
Acting before an emergency occurs	691	49.9 [46.8, 53.0]
Motivators^d^		
Increased likelihood of a local disaster	539	38.9 [36.0, 41.9]
Information about possible local disasters	498	36.0 [33.1, 38.9]
Personal experience with a disaster	325	23.5 [20.9, 26.0]
If I received a discount to buy basic supplies	199	14.4 [12.2, 16.5]
Information about consequences (losses)	176	12.7 [10.7, 14.8]
Nothing, I am already very prepared	278	20.1 [17.6, 22.5]
Barriers^e^		
Planning for the unknown is confusing	329	23.7 [21.2, 26.3]
I can’t afford to buy supplies	232	16.7 [14.4, 19.1]
I don’t think it is important where I live	151	10.9 [8.9, 12.8]
I don’t know where to begin or what to do	115	8.3 [6.4, 10.2]
Talking to my family about it is hard	73	5.3 [3.8, 6.8]

NOTE: CI = confidence interval.

a Weighted sample size of those who self-reported that they were *somewhat* or *very prepared*. ^b^Percentage was weighted to match the U.S. Current Population Survey proportions for sex, age, household income, race/ethnicity, household size, education level, census region, metro status, and whether or not a respondent had Internet access. ^c^Attitudes refer to the question “What does emergency preparedness for disasters and disease outbreaks mean to you?” ^d^Motivators refer to the question “What would make you more likely to prepare for emergencies?” ^e^Barriers refer to the question “What makes it difficult for you to prepare for emergencies?”

In the multivariable analyses (Table 3), respondents who perceived being prepared were more likely to report the following favorable attitudes toward preparedness: need for emergency supplies (adjusted odds ratio [AOR] = 1.25, 95% CI = [1.05, 1.50]), need to discuss emergency plans (AOR = 1.21, 95% CI = [1.02, 1.42]), and need to act before an emergency occurred (AOR = 1.35, 95% CI = [1.15, 1.59]), compared with those who perceived not being prepared.

**Table 3:** Perceived Preparedness and Characteristics Associated With Preparedness Attitudes, Motivating Factors, and Barriers Among Adults (⩾18 years)—*Styles* Survey, 2016

	Among Those Prepared			
Characteristics	*n* (Weighted)^a^	%^b^	AOR^c^	[95% CI]
Attitudes^d^				
Knowing what to do during an emergency	1,057	30.5	1.01	[0.82, 1.24]
Having the right emergency supplies	946	27.3	1.25*	[1.05, 1.50]
Being able to react to an unplanned event	903	26.1	1.19	[1.00, 1.41]
Discussing plans in case of emergencies	802	23.2	1.21*	[1.02, 1.42]
Preventing harm to myself and my family	784	22.7	1.18	[1.00, 1.39]
Acting before an emergency occurs	691	20.0	1.35*	[1.15, 1.59]
Motivators^e^				
Increased likelihood of a local disaster	539	15.6	0.73*	[0.62, 0.90]
Information about possible local disasters	498	14.4	0.90	[0.75, 1.05]
Personal experience with a disaster	325	9.4	0.84	[0.70, 1.02]
Nothing, I am already very prepared	278	8.0	9.92*	[6.70, 14.71]
If I received a discount to buy basic supplies	199	5.7	0.68*	[0.54, 0.84]
Information about consequences (losses)	176	5.1	0.64*	[0.50, 0.81]
Barriers^f^				
Planning for the unknown is confusing	329	9.5	0.63*	[0.53, 0.76]
I can’t afford to buy supplies	232	6.7	0.62*	[0.50, 0.76]
I don’t think it is important where I live	151	4.4	0.56*	[0.44, 0.72]
I don’t know where to begin or what to do	115	3.3	0.26*	[0.20, 0.35]
Talking to my family about it is hard	73	2.1	0.80	[0.54, 1.15]

NOTE: Data were only analyses for each major sociodemographic characteristic. Chi-square tests were used to calculate *p* values for unadjusted difference in the percent of those who reported being prepared and not prepared separately for each individual sociodemographic characteristic. AOR = adjusted odds ratio; CI = confidence interval.

a Weighted sample size of those who self-reported that they were somewhat or very prepared. ^b^Percentage is weighted to match the U.S. Current Population Survey proportions for sex, age, household income, race/ethnicity, household size, education level, census region, metro status, and whether or not a respondent had Internet access. ^c^Multiple logistic regression, adjusted for age, sex, race/ethnicity, and education. Those who said they were prepared were compared with those who were not at all or a little prepared. ^d^Attitudes refer to the question “What does emergency preparedness for disasters and disease outbreaks mean to you?” ^e^Motivators refer to the question “What would make you more likely to prepare for emergencies?” ^f^Barriers refer to the question “What makes it difficult for you to prepare for emergencies?”

* Significant at *p* < .05.

Adults who reported being prepared more likely reported that nothing would make them more prepared (AOR = 9.92, 95% CI = [6.70, 14.71]) compared with those who perceived not being prepared. Additional motivating competencies that were significant among those prepared include experiencing a local disaster (AOR = 0.73, 95% CI = [0.62, 0.90]), receiving a discount to buy basic supplies (AOR = 0.68, 95% CI = [0.54, 0.84]), and receiving information about the loss consequences of a disaster (AOR = 0.64, 95% CI = [0.50, 0.81]), compared with those who perceived not being prepared.

Adults who reported being prepared less likely reported confusion on how to plan for the unknown (AOR = 0.63, 95% CI = [0.53, 0.76]), inability to buy supplies (AOR = 0.62, 95% CI = [0.50, 0.76]), being unaware that it was important to prepare where they lived (AOR = 0.56, 95% CI = [0.44, 0.72]), and not knowing where to begin or what to do (AOR = 0.26, 95% CI = [0.20, 0.35]), compared with those who perceived not being prepared.

## Discussion

### Narrowing the Gap

This study found that approximately 40% of adults responding to the study survey reported being prepared for a disaster. Our findings are higher than those reported in another study using the Behavioral Risk Factor Surveillance System (BRFSS) between 2006 and 2010, which found that 25.3% of the population felt they were well prepared for an emergency (DeBastiani, Strine, Vagi, Barnett, & Kahn, 2015). It should be noted that the BRFSS survey used different questions to describe preparedness and that it collected data through landlines and cell phones rather than online. A multistate BRFSS analysis previously suggested that greater efforts are needed to increase accessibility of household preparedness materials and information to the Hispanic population and persons with language barriers (DeBastiani & Strine, 2012). Given the sizable investment in readiness (Petkiva et al., 2016; U.S. Department of Homeland Security, 2007), focusing on who to influence and how to do that most effectively (U. S. Department of Health and Human Services, 2017), holds the potential for more versatile planning to improve health outcomes (U.S. Department of Homeland Security, 2003). This could include focused campaigns in geographical areas of the country with a history of certain types of natural disasters, outbreaks, or populations at risk. This *Styles* study noted that more communication efforts could increase the reach of at-risk populations including younger adults (e.g., ⩽24 years of age), those with less education, unmarried individuals, and residents in the Northeast. Our study also found that 75% of adults participating in this survey reported knowing what to do during an emergency, although differences among those prepared and not prepared were found with regard to planning (e.g., emergency supplies) and information warning. Similarly, another study found that previous disaster experience was highly correlated to DPB (Najafi et al., 2015). However, another study indicated a mismatch between perceived preparedness and actual preparedness, suggesting the need for culturally appropriate efforts to reach potentially vulnerable subgroups (Ablah, Konda, & Kelley, 2009). The Ready campaign by the U.S. Department of Homeland Security underscores the importance of continued efforts to educate the public about the needs to develop an emergency plan, to communicate the plan with their family and friends, and to practice it (U.S. Department of Homeland Security, 2003). For this reason, the U.S. Department of Homeland Security, through local Citizen Corps Councils, coordinates with states, territories, tribes, and local communities across the country to assess knowledge of protective actions individuals should take to prepare for disasters (U.S. Department of Homeland Security, 2006).

### Attitudes, Motivators, and Barriers

The complexity of emergencies and the rise in the unknown necessitates a better understanding of what information people need to prepare before, during, and after a crisis. It is understandable that the attitudes of adults participating in this survey reveal confusion when it comes to emergency preparedness. This study found that emergency preparedness motivators include likelihood of a local disaster occurring, receipt of information about a possible local disaster, and having had a personal experience with a disaster. In an international study, researchers found that Iranian adults’ attitude and knowledge regarding earthquake preparedness affected their DPB (Ostad et al., 2012). Other factors such as educational status have also been associated with increasing odds of having emergency supplies (Eisenman et al., 2006) or for being prepared for an earthquake (Ostad et al., 2012). Substantial efforts are needed to raise familiarity of respondents to local hazards, increase their ability to obtain basic supplies, and raise their level of awareness about the consequences of an unplanned event. A previous study found that key motivating factors for household emergency preparedness were having children at home, advance notice of the disaster, knowing of others who experienced the hazard, experiencing the hazard firsthand, knowledge of preparation, and discussing disaster with friends and family (FEMA, 2013). A study by Strine, Neff, and Crawford (2013) identified the need to provide outreach to individuals with mental and physical health impairments and to include older adults. It is common for people to face uncertainty at the onset of the disaster, as situations are often uncertain and information is often unavailable, overly technical, and susceptible to competing interpretations (Kiel, 1995). Given the numerous health disparities among adults (Miller & Arquilla, 2008), and the challenges that go along with trying to cope with service disruption, emergency services, and health care systems, it is important that people understand what they can do to protect their health (Prue, 2016). Identifying commonly occurring natural disasters to guide preparedness campaigns and inform information dissemination strategies in advance is ideal. These efforts may also address preparedness barriers related to supplies and awareness about how to prepare for a disaster.

### Making Readiness Personal

Evidence suggests that individuals who have an understanding of the critical steps to take during an emergency and have the ability to take action to accomplish those steps are more prepared for disasters (Morton & Lurie, 2013). In the *Preparedness in America* report by the FEMA, respondents reported their main preparedness concerns as impact of disasters on family (including pets), fear of the unknown, ability to communicate with family and friends after a disaster, lack of resources (i.e., food, gas, alternative source of electricity), not surviving, and level of officials’ preparation (FEMA, 2013). As a result, FEMA continues to invest in ways to determine how self-efficacy informs community planning efforts and identified four preparedness profiles as an approach to tailoring messages and outreach campaigns (Butts et al., 2014). Additionally, increased applications of behavioral theories are used to inform community planning efforts to enhance community resilience (Adams et al., 2017; Paek, Hilyard, Frimuth, Barge, & Mindlin, 2010).

### Moving Forward

In order to track and assess household preparedness, surveys need to be administered more regularly if we are to improve our knowledge about attitudes, motivators, and barriers to action (Ablah et al., 2009). The use of Internet panel surveys such as *Styles* allows rapid query of perceived preparedness at the household level and further refinement of questions after being used in other surveys. Since health information technology continues to expand as a method of disseminating information through social networks, new communication strategies need to be adopted (i.e., alert systems) to notify communities before, during, and after emergencies occur. More efforts are needed to improve approaches to emergency communications through these types of social networks—Twitter, Facebook, and texting—and through the tools and data they employ and track (Baseman, Revere, Painter, & Oberle, 2016; Timler Boguaiak et al., 2016).

### Strengths and Limitations

Strengths of this study include the use of an Internet panel survey data to capture perception of household emergency preparedness by sociodemographic characteristics and assessment of household preparedness attitudes, motivators, and barriers among U.S. adults. *Styles* allows new survey questions to be added on novel and emerging public health threats, as well as on attitudes and behaviors related to changing policies related to preparedness, which could affect individual and community-level health. However, this study is subject to limitations. First, though *Styles* draws from an existing panel with a nationally representative sample, it does not recruit using population-based probability samples, which may have limited generalizability. Second, survey responses were self-reported, which could lead to reporting bias. Third, small sample sizes for some subpopulations resulted in less precise estimates that could not be presented (<50). Fourth, it is possible that a misinterpretation bias may have occurred based on how select questions were worded (e.g., *having the right emergency supplies* may be confused with *knowing which supplies are right*). Finally, although the results of the present study may help support the DPB theoretical model, more research is needed to determine if the measures are applicable in the United States. (Najafi et al., 2015).

## Conclusions and Future Study

This study found that adults who reported being prepared were more likely to have the right emergency supplies, discuss plans in case of emergencies, and act before an emergency occurs. Despite significant investment in preparedness, less than half of the participating adults perceived that they are prepared for an emergency, and one quarter stated that they do not know what to do during an emergency. To promote higher levels of preparedness, coordinated efforts using ongoing and improved survey instruments that collect more detailed information about household and community preparedness are needed. The results of this study may inform the development of future interventions aimed at improving perceived control over DPB. Investments should be informed by jurisdictional need and available resources. Public health planners should remain mindful of the disparities and barriers that exist between people who reported being prepared and those who did not in surveys of this nature. Public health emergency managers should consider using multiple information delivery networks to communicate information with diverse groups. Continued efforts for national, state, and local education of individuals and communities should ensure that the steps, required tools, and appropriate actions are taken before a disaster occurs.
